# 1-(3-Chloro-4-fluoro­phen­yl)-5-(2-diazo­acet­yl)-4-phenyl­pyrrolidin-2-one

**DOI:** 10.1107/S1600536810024657

**Published:** 2010-07-24

**Authors:** Jayanta Kumar Ray, Pranab Haldar, M. Canle L., M. I. Fernández P., J. A. Santaballa

**Affiliations:** a Department of Chemistry, Indian Institute of Technology, Kharagpur 721 302, India; b Departamento de Química Física e Enxeñería Química I, Facultade de Ciencias, Universidade da Coruña, Rúa Alejandro de la Sota 1, E-15008 A Coruña, Spain

## Abstract

In the title compound, C_18_H_13_ClFN_3_O_2_, the pyrrolidine ring adopts an envelope conformation and the planar part is rotated by 4.3 (6)° from the plane of the benzene ring and is almost perperdicular both to the diazo­acetyl unit [dihedral angle = 78.93 (7)°] and the phenyl ring [dihedral angle = 86.07 (7)°]. In the crystal, mol­ecules are linked into a three-dimensional framework by C—H⋯O inter­actions. The mol­ecular conformation is stabilized by an intra­molecular C—H⋯O hydrogen bond.

## Related literature

For synthetic methods, see: (Ray *et al.* 1994 ▶, 1998 ▶). For bond-length data, see: Allen (2002 ▶). For related compound see: Ray *et al.* (2004 ▶). For puckering parameters, see: Cremer & Pople (1975 ▶). For hydrogen bonding, see: Desiraju (2005 ▶). For a description of the Cambridge Structural Database, see: Allen (2002 ▶).
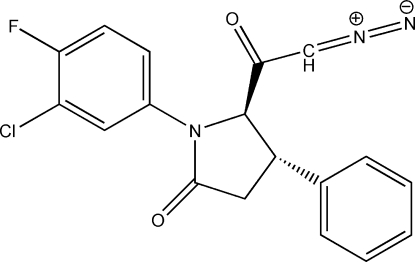


## Experimental

### 

#### Crystal data

C_18_H_13_ClFN_3_O_2_
*M* *_r_* = 357.76
Monoclinic, 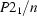

*a* = 10.3498 (3) Å
*b* = 9.2252 (3) Å
*c* = 17.1639 (5) Å
β = 91.088 (2)°
*V* = 1638.50 (9) Å^3^
*Z* = 4
Mo *K*α radiation
μ = 0.26 mm^−1^
*T* = 100 K
0.42 × 0.38 × 0.21 mm

#### Data collection

Bruker APEXII area-detector diffractometer
Absorption correction: multi-scan (*SADABS*; Bruker, 2003 ▶) *T*_min_ = 0.901, *T*_max_ = 0.952
16359 measured reflections
4084 independent reflections
3597 reflections with *I* > 2σ(*I*)
*R*_int_ = 0.021

#### Refinement

*R*[*F* ^2^ > 2σ(*F* ^2^)] = 0.035
*wR*(*F* ^2^) = 0.123
*S* = 0.90
4084 reflections
278 parameters
All H-atom parameters refined
Δρ_max_ = 0.42 e Å^−3^
Δρ_min_ = −0.33 e Å^−3^

### 

Data collection: *APEX2* (Bruker, 2005 ▶); cell refinement: *SAINT* (Bruker, 2004 ▶); data reduction: *SAINT* and *XPREP* (Bruker, 2003 ▶); program(s) used to solve structure: *SHELXS97* (Sheldrick, 2008 ▶); program(s) used to refine structure: *SHELXL97* (Sheldrick, 2008 ▶); molecular graphics: *PLUTON* (Spek, 2009 ▶); software used to prepare material for publication: *SHELXL97*.

## Supplementary Material

Crystal structure: contains datablocks global, I. DOI: 10.1107/S1600536810024657/bx2286sup1.cif

Structure factors: contains datablocks I. DOI: 10.1107/S1600536810024657/bx2286Isup2.hkl

Additional supplementary materials:  crystallographic information; 3D view; checkCIF report

## Figures and Tables

**Table 1:** Hydrogen-bond geometry (Å, °)

*D*—H⋯*A*	*D*—H	H⋯*A*	*D*⋯*A*	*D*—H⋯*A*
C4—H4⋯O1^i^	0.946 (18)	2.589 (18)	3.3445 (19)	137.1 (14)
C10—H10⋯O2^ii^	0.955 (16)	2.340 (16)	3.2393 (15)	156.8 (13)
C12—H12⋯O1	0.922 (17)	2.211 (16)	2.8328 (17)	124.1 (13)
C12—H12⋯O1^iii^	0.922 (17)	2.505 (16)	3.2586 (17)	139.1 (13)
C16—H16⋯O2^ii^	0.946 (18)	2.460 (18)	3.3403 (16)	154.9 (15)
C18—H18⋯O2^ii^	0.944 (18)	2.482 (19)	3.2320 (16)	136.4 (15)

Symmetry codes: (i) 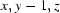; (ii) 
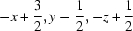
; (iii) 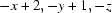.

**Fig. 1.**
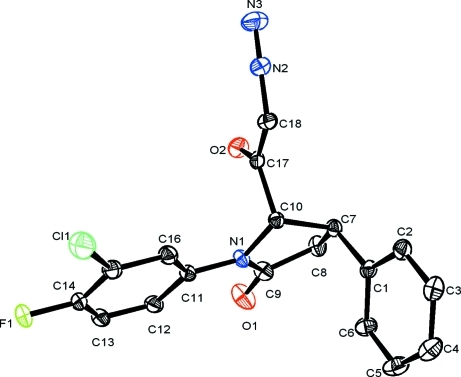
View of the title compound showing the atomic numbering and 50% probability displacement ellipsoids. H atoms have been omitted for clarity.

**Fig. 2.**
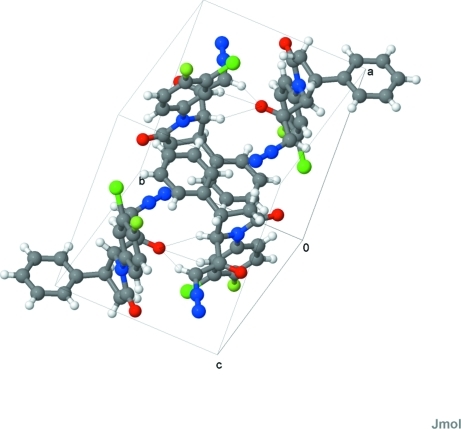
Part of the crystal structure showing carbonyl oxygen O2 interacts with three H atoms.

## supplementary crystallographic information

### Comment

A view of the molecule with numbering scheme is shown in Figure 1. In the title compounnd (I) , bond distances and angles are within normal ranges (Allen, 2002) .The atoms of the chain contaning the diazo group are planar [max deviation 0.339 (9)Å for N1].The pyrrolidin ring adopts an envelope conformation with puckering parameters q2 = 0.2816 (12) Å and φ_2_ = 115.6 (2)°(Cremer & Pople, 1975). The 3-chloro-4-fluoro-benzene ring and the best plane of pyrrolidin ring defined by C10\N1\C9\C8 atoms are almost coplanar [4.20 (7)°]. The phenyl ring and the acetyl group O2/C10/C17/C18 fragment (r.m.s. deviation 0.023 Å)  are almost perpendicular  to  plane defined by the N1/C8/C9/C10/C11/C12/C13/C14/C15/C16 atoms (r.m.s. deviation 0.038 Å) [87.07 (5); 88.46 (3)° respectively]. In the crystal molecules are linked by C—H···O interactions [C···O range 3.2320 (16)-3.3445 (19) Å; C—H···O range 136-157°] and these link the molecules into a three-dimensional framework. The molecular conformation is stabilized by one intramolecular C—H···O hydrogen bond (Table 1). As shown in Figure 2, carbonyl oxygen O2 interacts with three H atoms (C10—H10···O2, C16—H18···O2, and C18—H18···O2), which regarding crystal packing could be classified as supportive (Desiraju, 2005).

### Experimental

The title compound was synthesized from the corresponding γ-lactam carboxylic acid which, in turn, was prepared following the general method (Ray *et al.* 1994, 1998) developed in our laboratory, through the reaction of its acid chloride with diazomethane. Single crystal was grown by dissolving the compound in mixture (n-hexane-ethylacetate) solvent and then by slow evaporation technique at room temperature. It is a yellow colour solid; m.p. 407–409 K (n-hexane-ethylacetate).

### Refinement

Hydrogen atoms were found in subsequent difference Fourier maps and included in observed positions and refined as free isotropic atoms.

### Crystal data

**Table:**

C_18_H_13_ClFN_3_O_2_	*F*(000) = 736
*M**_r_* = 357.76	*D*_x_ = 1.450 Mg m^−^^3^
Monoclinic, *P*2_1_/*n*	Melting point: 408 K
Hall symbol: -P 2yn	Mo *K*α radiation, λ = 0.71073 Å
*a* = 10.3498 (3) Å	Cell parameters from 7474 reflections
*b* = 9.2252 (3) Å	θ = 2.5–28.3°
*c* = 17.1639 (5) Å	µ = 0.26 mm^−^^1^
β = 91.088 (2)°	*T* = 100 K
*V* = 1638.50 (9) Å^3^	Block, yellow
*Z* = 4	0.42 × 0.38 × 0.21 mm

### Data collection

**Table:**

Bruker APEXII area-detector diffractometer	4084 independent reflections
Radiation source: fine-focus sealed tube	3597 reflections with *I* > 2σ(*I*)
graphite	*R*_int_ = 0.021
phi and ω scans	θ_max_ = 28.3°, θ_min_ = 2.5°
Absorption correction: multi-scan (*SADABS*; Bruker, 2003)	*h* = −13→13
*T*_min_ = 0.901, *T*_max_ = 0.952	*k* = −12→12
16359 measured reflections	*l* = −22→22

### Refinement

**Table:**

Refinement on *F*^2^	Primary atom site location: structure-invariant direct methods
Least-squares matrix: full	Secondary atom site location: difference Fourier map
*R*[*F*^2^ > 2σ(*F*^2^)] = 0.035	Hydrogen site location: inferred from neighbouring sites
*wR*(*F*^2^) = 0.123	All H-atom parameters refined
*S* = 0.90	*w* = 1/[σ^2^(*F*_o_^2^) + (0.1*P*)^2^ + 0.4639*P*] where *P* = (*F*_o_^2^ + 2*F*_c_^2^)/3
4084 reflections	(Δ/σ)_max_ = 0.001
278 parameters	Δρ_max_ = 0.42 e Å^−^^3^
0 restraints	Δρ_min_ = −0.33 e Å^−^^3^

### Special details

**Table:**

Experimental. Data was collected using a X8 *APEX* II BRUKER-Nonius diffractometer equipped with an KYROFLEX low-temperature apparatus operating at 100 K. A suitable crystal was chosen and mounted on Mitegen MicroMount (radiation-hard polymer).Data were measured using omega scans of 0.5° per frame for 10 s, such that a total of 1280 frames were collected in a optimized strategy and with a final resolution of 0.75 Å. Data integration and reduction was performed using the Apex2 (Bruker Nonius, 2005) suite software.Absorption corrections were applied using *SADABS* (2004) of the suite software.The structures are solved by the direct method using the *SHELX97* program and refined by least squares method on F2 *SHELXL97*, incorporated in the Apex2 suite software.
Geometry. All e.s.d.'s (except the e.s.d. in the dihedral angle between two l.s. planes) are estimated using the full covariance matrix. The cell e.s.d.'s are taken into account individually in the estimation of e.s.d.'s in distances, angles and torsion angles; correlations between e.s.d.'s in cell parameters are only used when they are defined by crystal symmetry. An approximate (isotropic) treatment of cell e.s.d.'s is used for estimating e.s.d.'s involving l.s. planes.
Refinement. Refinement of *F*^2^ against ALL reflections. The weighted *R*-factor *wR* and goodness of fit *S* are based on *F*^2^, conventional *R*-factors *R* are based on *F*, with *F* set to zero for negative *F*^2^. The threshold expression of *F*^2^ > σ(*F*^2^) is used only for calculating *R*-factors(gt) *etc*. and is not relevant to the choice of reflections for refinement. *R*-factors based on *F*^2^ are statistically about twice as large as those based on *F*, and *R*- factors based on ALL data will be even larger.All non-hydrogen atoms were refined anisotropically. Hydrogen were found in subsequent difference Fourier maps and included in observed positions and refined as free isotropic atoms.

### Fractional atomic coordinates and isotropic or equivalent isotropic displacement parameters (Å^2^)

**Table:**

	*x*	*y*	*z*	*U*_iso_*/*U*_eq_	
Cl1	0.42826 (3)	0.11958 (5)	0.07084 (2)	0.03671 (13)	
F1	0.46336 (8)	0.32525 (11)	−0.05664 (5)	0.0361 (2)	
N1	0.88460 (9)	0.29021 (10)	0.14689 (5)	0.0156 (2)	
N2	0.67780 (10)	0.24925 (13)	0.39276 (6)	0.0236 (2)	
N3	0.63827 (13)	0.30402 (17)	0.44487 (7)	0.0385 (3)	
O1	1.02767 (10)	0.45547 (12)	0.09513 (6)	0.0329 (2)	
O2	0.80348 (9)	0.41556 (9)	0.28615 (5)	0.0236 (2)	
C1	1.07129 (10)	0.04602 (13)	0.21682 (6)	0.0167 (2)	
C2	1.04967 (11)	−0.07912 (14)	0.26035 (7)	0.0200 (2)	
H2	1.0083 (16)	−0.0735 (18)	0.3097 (10)	0.025 (4)*	
C3	1.08407 (12)	−0.21444 (14)	0.23217 (8)	0.0230 (3)	
H3	1.0708 (17)	−0.2969 (19)	0.2610 (10)	0.031 (4)*	
C4	1.14189 (12)	−0.22676 (15)	0.16014 (8)	0.0260 (3)	
H4	1.1586 (16)	−0.320 (2)	0.1404 (10)	0.031 (4)*	
C5	1.16535 (13)	−0.10307 (16)	0.11693 (8)	0.0269 (3)	
H5	1.2026 (18)	−0.112 (2)	0.0673 (11)	0.040 (5)*	
C6	1.13024 (12)	0.03259 (14)	0.14473 (7)	0.0221 (3)	
H6	1.1435 (15)	0.1177 (18)	0.1141 (10)	0.023 (4)*	
C7	1.01876 (10)	0.18845 (13)	0.24651 (6)	0.0162 (2)	
H7	1.0221 (15)	0.1892 (17)	0.3046 (9)	0.022 (4)*	
C8	1.08093 (12)	0.32552 (14)	0.21426 (7)	0.0207 (2)	
H8A	1.1684 (17)	0.3136 (19)	0.2006 (10)	0.028 (4)*	
H8B	1.0804 (16)	0.4057 (18)	0.2524 (10)	0.025 (4)*	
C9	0.99853 (12)	0.36796 (13)	0.14439 (7)	0.0202 (2)	
C10	0.87542 (10)	0.20363 (12)	0.21788 (6)	0.0138 (2)	
H10	0.8420 (14)	0.1091 (15)	0.2073 (8)	0.010 (3)*	
C11	0.77808 (11)	0.30129 (12)	0.09402 (6)	0.0157 (2)	
C12	0.78022 (12)	0.39482 (13)	0.02979 (7)	0.0199 (2)	
H12	0.8530 (15)	0.4502 (17)	0.0199 (8)	0.016 (3)*	
C13	0.67333 (13)	0.40285 (15)	−0.02029 (7)	0.0244 (3)	
H13	0.6734 (18)	0.477 (2)	−0.0662 (11)	0.037 (5)*	
C14	0.56682 (12)	0.31791 (16)	−0.00738 (7)	0.0248 (3)	
C15	0.56378 (11)	0.22465 (15)	0.05545 (7)	0.0223 (3)	
C16	0.66909 (11)	0.21630 (14)	0.10649 (7)	0.0190 (2)	
H16	0.6620 (16)	0.1485 (19)	0.1490 (10)	0.029 (4)*	
C17	0.79785 (10)	0.28288 (12)	0.27989 (6)	0.0161 (2)	
C18	0.72863 (11)	0.18918 (14)	0.33019 (6)	0.0185 (2)	
H18	0.7186 (17)	0.088 (2)	0.3236 (10)	0.034 (5)*	

### Atomic displacement parameters (Å^2^)

**Table:**

	*U*^11^	*U*^22^	*U*^33^	*U*^12^	*U*^13^	*U*^23^
Cl1	0.01855 (18)	0.0601 (3)	0.0313 (2)	−0.01115 (14)	−0.00341 (13)	−0.00666 (15)
F1	0.0259 (4)	0.0618 (6)	0.0201 (4)	0.0150 (4)	−0.0103 (3)	−0.0053 (4)
N1	0.0156 (4)	0.0170 (5)	0.0141 (4)	−0.0034 (3)	−0.0011 (3)	0.0027 (3)
N2	0.0196 (5)	0.0311 (6)	0.0202 (5)	0.0038 (4)	0.0024 (4)	0.0001 (4)
N3	0.0342 (7)	0.0559 (9)	0.0257 (6)	0.0084 (6)	0.0064 (5)	−0.0094 (5)
O1	0.0330 (5)	0.0366 (6)	0.0290 (5)	−0.0187 (4)	−0.0038 (4)	0.0127 (4)
O2	0.0291 (5)	0.0154 (4)	0.0264 (4)	0.0025 (3)	0.0014 (3)	−0.0023 (3)
C1	0.0117 (5)	0.0201 (6)	0.0183 (5)	0.0002 (4)	−0.0008 (4)	−0.0009 (4)
C2	0.0164 (5)	0.0229 (6)	0.0206 (5)	0.0019 (4)	0.0003 (4)	0.0020 (4)
C3	0.0193 (5)	0.0196 (6)	0.0300 (6)	0.0008 (4)	−0.0007 (5)	0.0021 (5)
C4	0.0197 (6)	0.0239 (6)	0.0345 (7)	0.0013 (5)	0.0022 (5)	−0.0081 (5)
C5	0.0222 (6)	0.0320 (7)	0.0267 (6)	−0.0004 (5)	0.0082 (5)	−0.0066 (5)
C6	0.0188 (5)	0.0251 (6)	0.0228 (5)	−0.0019 (4)	0.0054 (4)	0.0001 (5)
C7	0.0139 (5)	0.0189 (5)	0.0156 (5)	−0.0003 (4)	−0.0013 (4)	0.0002 (4)
C8	0.0166 (5)	0.0213 (6)	0.0241 (6)	−0.0049 (4)	−0.0038 (4)	0.0008 (4)
C9	0.0198 (5)	0.0201 (6)	0.0205 (5)	−0.0065 (4)	−0.0010 (4)	−0.0001 (4)
C10	0.0139 (5)	0.0137 (5)	0.0139 (5)	−0.0005 (4)	0.0002 (4)	0.0015 (4)
C11	0.0166 (5)	0.0165 (5)	0.0140 (5)	0.0015 (4)	−0.0010 (4)	−0.0017 (4)
C12	0.0243 (6)	0.0195 (6)	0.0158 (5)	0.0031 (4)	0.0012 (4)	0.0006 (4)
C13	0.0299 (6)	0.0278 (7)	0.0153 (5)	0.0110 (5)	−0.0011 (4)	0.0009 (4)
C14	0.0208 (6)	0.0377 (7)	0.0158 (5)	0.0118 (5)	−0.0048 (4)	−0.0069 (5)
C15	0.0157 (5)	0.0324 (7)	0.0186 (5)	0.0004 (5)	−0.0007 (4)	−0.0059 (5)
C16	0.0168 (5)	0.0238 (6)	0.0162 (5)	−0.0012 (4)	−0.0006 (4)	−0.0014 (4)
C17	0.0149 (5)	0.0171 (5)	0.0161 (5)	0.0028 (4)	−0.0024 (4)	0.0002 (4)
C18	0.0186 (5)	0.0203 (6)	0.0168 (5)	0.0027 (4)	0.0033 (4)	0.0001 (4)

### Geometric parameters (Å, °)

**Table:**

Cl1—C15	1.7294 (13)	C6—H6	0.956 (16)
F1—C14	1.3534 (13)	C7—C8	1.5275 (16)
N1—C9	1.3815 (14)	C7—C10	1.5602 (15)
N1—C11	1.4182 (13)	C7—H7	0.996 (16)
N1—C10	1.4616 (13)	C8—C9	1.5098 (16)
N2—N3	1.1120 (16)	C8—H8A	0.946 (17)
N2—C18	1.3262 (15)	C8—H8B	0.988 (16)
O1—C9	1.2114 (15)	C10—C17	1.5311 (15)
O2—C17	1.2300 (14)	C10—H10	0.954 (14)
C1—C2	1.3954 (16)	C11—C16	1.3937 (16)
C1—C6	1.3955 (15)	C11—C12	1.4005 (15)
C1—C7	1.5140 (16)	C12—C13	1.3899 (17)
C2—C3	1.3877 (17)	C12—H12	0.929 (15)
C2—H2	0.959 (17)	C13—C14	1.374 (2)
C3—C4	1.3884 (18)	C13—H13	1.047 (19)
C3—H3	0.919 (18)	C14—C15	1.3803 (19)
C4—C5	1.385 (2)	C15—C16	1.3874 (15)
C4—H4	0.941 (18)	C16—H16	0.964 (17)
C5—C6	1.3901 (18)	C17—C18	1.4245 (16)
C5—H5	0.945 (19)	C18—H18	0.944 (19)
			
C9—N1—C11	126.61 (9)	O1—C9—N1	126.27 (11)
C9—N1—C10	112.25 (9)	O1—C9—C8	125.65 (11)
C11—N1—C10	120.71 (9)	N1—C9—C8	108.08 (10)
N3—N2—C18	177.31 (15)	N1—C10—C17	111.22 (9)
C2—C1—C6	118.59 (11)	N1—C10—C7	103.60 (8)
C2—C1—C7	118.39 (10)	C17—C10—C7	109.39 (8)
C6—C1—C7	122.78 (11)	N1—C10—H10	111.7 (8)
C3—C2—C1	120.85 (11)	C17—C10—H10	112.0 (8)
C3—C2—H2	118.6 (10)	C7—C10—H10	108.5 (8)
C1—C2—H2	120.5 (10)	C16—C11—C12	119.50 (11)
C2—C3—C4	120.12 (12)	C16—C11—N1	118.87 (10)
C2—C3—H3	121.0 (11)	C12—C11—N1	121.63 (10)
C4—C3—H3	118.9 (11)	C13—C12—C11	119.69 (12)
C5—C4—C3	119.49 (12)	C13—C12—H12	119.8 (9)
C5—C4—H4	121.6 (11)	C11—C12—H12	120.5 (9)
C3—C4—H4	118.8 (11)	C14—C13—C12	120.10 (11)
C4—C5—C6	120.55 (12)	C14—C13—H13	120.6 (10)
C4—C5—H5	119.6 (12)	C12—C13—H13	119.3 (10)
C6—C5—H5	119.8 (12)	F1—C14—C13	119.90 (12)
C5—C6—C1	120.39 (12)	F1—C14—C15	119.32 (12)
C5—C6—H6	120.6 (10)	C13—C14—C15	120.78 (11)
C1—C6—H6	118.9 (10)	C14—C15—C16	119.91 (12)
C1—C7—C8	116.10 (10)	C14—C15—Cl1	119.95 (9)
C1—C7—C10	108.50 (9)	C16—C15—Cl1	120.14 (10)
C8—C7—C10	102.48 (9)	C15—C16—C11	120.01 (11)
C1—C7—H7	109.7 (9)	C15—C16—H16	116.3 (10)
C8—C7—H7	110.5 (9)	C11—C16—H16	123.7 (10)
C10—C7—H7	109.2 (9)	O2—C17—C18	125.07 (11)
C9—C8—C7	105.49 (9)	O2—C17—C10	120.74 (10)
C9—C8—H8A	111.2 (10)	C18—C17—C10	114.08 (10)
C7—C8—H8A	113.9 (11)	N2—C18—C17	116.68 (11)
C9—C8—H8B	108.8 (10)	N2—C18—H18	117.7 (11)
C7—C8—H8B	111.8 (10)	C17—C18—H18	125.6 (11)
H8A—C8—H8B	105.6 (15)		
			
C6—C1—C2—C3	−1.00 (17)	C1—C7—C10—C17	145.75 (9)
C7—C1—C2—C3	173.48 (10)	C8—C7—C10—C17	−90.94 (10)
C1—C2—C3—C4	0.59 (18)	C9—N1—C11—C16	−178.55 (11)
C2—C3—C4—C5	0.27 (19)	C10—N1—C11—C16	−6.68 (16)
C3—C4—C5—C6	−0.7 (2)	C9—N1—C11—C12	1.68 (17)
C4—C5—C6—C1	0.28 (19)	C10—N1—C11—C12	173.55 (10)
C2—C1—C6—C5	0.56 (17)	C16—C11—C12—C13	0.61 (17)
C7—C1—C6—C5	−173.66 (11)	N1—C11—C12—C13	−179.62 (10)
C2—C1—C7—C8	160.07 (10)	C11—C12—C13—C14	−0.89 (18)
C6—C1—C7—C8	−25.70 (15)	C12—C13—C14—F1	−179.48 (11)
C2—C1—C7—C10	−85.24 (12)	C12—C13—C14—C15	0.51 (19)
C6—C1—C7—C10	88.99 (12)	F1—C14—C15—C16	−179.86 (11)
C1—C7—C8—C9	92.73 (11)	C13—C14—C15—C16	0.15 (19)
C10—C7—C8—C9	−25.32 (12)	F1—C14—C15—Cl1	−0.39 (17)
C11—N1—C9—O1	−2.4 (2)	C13—C14—C15—Cl1	179.62 (10)
C10—N1—C9—O1	−174.83 (13)	C14—C15—C16—C11	−0.42 (18)
C11—N1—C9—C8	177.36 (10)	Cl1—C15—C16—C11	−179.89 (9)
C10—N1—C9—C8	4.91 (13)	C12—C11—C16—C15	0.04 (18)
C7—C8—C9—O1	−166.45 (13)	N1—C11—C16—C15	−179.74 (10)
C7—C8—C9—N1	13.82 (13)	N1—C10—C17—O2	−34.57 (13)
C9—N1—C10—C17	96.37 (11)	C7—C10—C17—O2	79.26 (12)
C11—N1—C10—C17	−76.58 (12)	N1—C10—C17—C18	149.15 (9)
C9—N1—C10—C7	−21.03 (12)	C7—C10—C17—C18	−97.02 (11)
C11—N1—C10—C7	166.01 (9)	O2—C17—C18—N2	−7.17 (17)
C1—C7—C10—N1	−95.58 (10)	C10—C17—C18—N2	168.93 (10)
C8—C7—C10—N1	27.74 (11)		

### Hydrogen-bond geometry (Å, °)

**Table:**

*D*—H···*A*	*D*—H	H···*A*	*D*···*A*	*D*—H···*A*
C4—H4···O1^i^	0.946 (18)	2.589 (18)	3.3445 (19)	137.1 (14)
C10—H10···O2^ii^	0.955 (16)	2.340 (16)	3.2393 (15)	156.8 (13)
C12—H12···O1	0.922 (17)	2.211 (16)	2.8328 (17)	124.1 (13)
C12—H12···O1^iii^	0.922 (17)	2.505 (16)	3.2586 (17)	139.1 (13)
C16—H16···O2^ii^	0.946 (18)	2.460 (18)	3.3403 (16)	154.9 (15)
C18—H18···O2^ii^	0.944 (18)	2.482 (19)	3.2320 (16)	136.4 (15)

Symmetry codes: (i) *x*, *y*−1, *z*; (ii) −*x*+3/2, *y*−1/2, −*z*+1/2; (iii) −*x*+2, −*y*+1, −*z*.
